# LAMP Reaction in Plant Disease Surveillance: Applications, Challenges, and Future Perspectives

**DOI:** 10.3390/life14121549

**Published:** 2024-11-26

**Authors:** Chiara Aglietti, Alessandra Benigno, Santa Olga Cacciola, Salvatore Moricca

**Affiliations:** 1 Department of Agricultural, Food, Environmental and Forestry Science and Technology (DAGRI), Plant Pathology and Entomology Section, University of Florence, Piazzale delle Cascine 28, 50144 Florence, Italy; alessandra.benigno@unifi.it (A.B.); salvatore.moricca@unifi.it (S.M.); 2 Department of Agriculture, Food and Environment, University of Catania, 95123 Catania, Italy; olgacacciola@unict.it

**Keywords:** loop-mediated isothermal amplification, phytosanitary checks, pathogen detection, global trade, climate change, invasive species, diagnostics, biosecurity, point of care (POC)

## Abstract

Movements of plant pathogenic microorganisms in uncontaminated areas occur today at an alarming rate, driven mainly by global trade and climate change. These invaders can trigger new disease outbreaks able to impact the biodiversity and economies of vast territories and affect a variety of ecosystem services. National and supranational regulatory deficiencies, such as inadequate quarantine measures and ineffective early pathogen detection at ports of entry, exacerbate the issue. Thus, there is an urgent need for accurate and rapid diagnostic tools to intercept invasive and nonindigenous plant pathogens. The LAMP (Loop-mediated isothermal AMPlification) technique is a robust, flexible tool representing a significant advance in point-of-care (POC) diagnostics. Its user-friendliness and sensitivity offer a breakthrough in phytosanitary checks at points of entry (harbors and airports), for disease and pest surveillance at vulnerable sites (e.g., nurseries and wood-processing and storage facilities), and for territorial monitoring of new disease outbreaks. This review highlights the strengths and weaknesses of LAMP, emphasizing its potential to revolutionize modern plant disease diagnostics.

## 1. Introduction

### 1.1. Alien Plant Pathogens: At the Roots of the Problem

The problem of Invasive Alien Pathogens and Pests (IAPPs) in agriculture and forestry can be defined as a side effect of the development of human society [1,2]. In fact, starting from the dawn of human civilization, with the end of the last ice age and the discovery of agriculture, humans began to foster pest movement through nomadic agriculture. In the following millennia, humans introduced species from foreign territories, making life easier for populations, facilitating food sustenance, and improving health and life expectancy [3,4,5]. China has a long and documented history, dating back over 6000 years, of the introduction of plants and animals along ancient trade routes (just remember the silk road). This process received a significant boost at the time of major geographic explorations, which led to the colonization of undiscovered lands. Many new plant species were discovered and imported to the Old Continent from the Americas. The flow was bidirectional: the first European settlers brought to the New Continent everything they deemed necessary (i.e., medicinal herbs, garden and flower plants, pets, etc.) for their immediate survival in an unknown environment that they feared was hostile. The pattern of introducing plants into North America was so massive that most of the species used today in agriculture, especially in horticulture, are not native to the continent. Plant pathogenic microbes “hitchhiked” from continent to continent, concealed inside infected plant tissues and goods, reaching undetected new, uncontaminated territories [6,7,8,9]. This process was given a boost in the modern era as human society transformed drastically, becoming more and more urbanized and industrialized.

### 1.2. Modern Drivers of Pathogen Invasions: Call for Early Detection

With technological progress, commercial exchanges have burgeoned, magnifying man’s ability to move goods and foodstuffs and enhancing human mobility (for work, tourism, etc.). Concurrently, modern technological progress has heightened anthropogenic disturbance of agricultural and natural systems through deforestation, crop conversions, biodiversity losses, changes in land use, land movement, environmental alterations (e.g., pesticide poisoning and pollution), etc. [10,11,12,13]. All these negative impacts are harbingers of pest invasions and phytosanitary problems, often resulting in “cascade” effects. For example, the increase in CO_2_ in the atmosphere is a major driver of global warming, which, in turn, facilitates the dispersal and invasion of new territories by thermophilic and/or thermotolerant plant pathogenic microbes. Given this alarming scenario, the early diagnosis of plant diseases has become an issue of paramount importance—even more so if achieved prior to the onset of symptoms (i.e., at a latent/pre-symptomatic stage) [14,15]. The opportunity to rely on efficient and simple diagnostic methods plays a key role in the decision-making process (e.g., eradication and containment measures), mainly with the aim of preventing or limiting the introduction and spread of harmful pathogens and pests into uncontaminated areas, where they could have severe economic and social consequences [5,16,17]. Concerning notifiable organisms, measures are usually needed to prevent their incursion into a new environment, often resulting in the destruction of infested consignments. For non-notifiable pests, actions are more often targeted at mitigating their impact [18]. In both cases, the faster the disease agent is identified, the faster decisions on its management can be taken, allowing for the implementation of prompt and more effective control strategies [18,19].

### 1.3. The Socio-Economic Impact of Plant Disease Invasions

Nowadays, the threat posed by IAPPs is heightened [3,4,5] due to microbial adaptation to new ecosystems brought about by trade and transportation and by climate change [6,7,8,9]. Due to these factors, as well as the other causes mentioned in the previous paragraph, the rate of introduction of foreign pathogens and pests into uncontaminated areas is occurring at an unprecedented rate in human history. In the Anthropocene, IAPPs threaten not only forest and agricultural crops but also the ecological and socio-economic systems of many countries all over the world [20]. In some situations, the damage can be very considerable and can give rise to negative spillovers in related sectors, such as landscape and tourism. For example, the Bayoud disease of the date palm caused by fungal pathogen *Fusarium oxysporum* f. sp. *albedinis* in Algeria and Morocco, in addition to severely reducing the production of dates, a primary economic source for those countries, has literally devastated many oases that previously generated important tourism income. In Italy (Apulia region), the severe branch desiccation and rapid death of centennial and monumental olive trees caused by bacterium *Xylella fastidiosa* have practically eliminated the production of olives and olive oil and destroyed an important peri-urban landscape characterized by tourist use. Quantifying the damage exotic agents of damage are causing is complex, and only a few studies have calculated the cost of multiple alien disease introduction either on a regional or global scale [2]. According to Ghelardini et al. [12] and Brownlie et al. [13], losses and control costs associated with plant diseases introduced from abroad reach about USD 21 billion annually. With regard to forest trees, Pimentel et al. [21] calculated that approximately USD 2.1 billion of forest products are lost each year due to alien forest pathogens in the USA. Apart from production losses, the full economic costs of invasions include negative side effects on the trade of forest products and plants; control expenses due to inspections, monitoring, prevention, and response; and ecological and environmental impacts on ecosystems [3,6,18].

### 1.4. Shortcomings of Traditional Prevention and Control Measures

According to Leung et al. [14] and Olson et al. [15], prevention and control are the two basic ways through which benefits able to far exceed the potential economic and ecological costs of an invasion can be obtained. While in some countries (e.g., Australia, New Zealand, Canada, and USA) there are strict biosecurity regulations on plant pests (e.g., the importation of soil is forbidden), Europe permits the importation of plants rooted in soil from outside the EU when they are officially declared free from harmful organisms (phytosanitary certificate or plant passport within the EU territory) and show no evident sign of infestation or disease [11]. However, inspections usually focus on well-known pests that are supposed to affect economically important crops and are often conducted in a short time, usually limited to visual examination of the aerial plant parts. Hence, these superficial inspection and surveillance procedures, often accompanied by classic diagnostic methods, may fail to detect regulated and non-regulated pests and pathogens, especially if they occur at an asymptomatic stage or if incipient symptoms are limited to the roots, which are often not inspected, allowing diseases to be introduced and spread undetected [1,10]. For example, Ruano-Rota et al. [22] demonstrated the limits of classical diagnostic methods for the detection of *Rosellinia necatrix* in avocado samples. These authors compared the results obtained by applying either classical (symptom observation and isolation on culture media) or molecular diagnostic tools to samples. Classical diagnostic techniques resulted in lower sensitivity, sometimes yielding negative results, even in the presence of evident symptomatology. Culture isolations did not permit the detection of the pathogen in some root samples with clear symptoms of *Rosellinia necatrix* infection. In addition, the same authors found that soil samples, where the inoculum is assumed to be very low, tested negative under classical techniques, whereas the same samples tested positive only under molecular-based assays. Narayanasamy [23] claimed that the identification of fungal plant pathogens up to the generic/species level and even at the *formae speciales* level is possible with classical methods if an accurate analysis of morphological characters and reproductive organs of the pathogen is carried out. However, culturing the disease agent is not always straightforward, since it requires an equipped lab and expert personnel and is, above all, time-consuming, especially if inoculation tests are to be performed to identify specialized forms of the pathogen. Furthermore, laboratory cultivation of the etiological agent is impossible if it is a biotrophic microorganism. For all these reasons, more effective, culture-independent, and early detection methods are highly in demand to unequivocally diagnose plant pathogens, even at pre-symptomatic stages [24].

### 1.5. The Step Forward of Molecular Tools

Among tools optimized and developed for plant pathogen detection, DNA- and RNA-based methods like polymerase chain reaction (PCR) and quantitative PCR (qPCR) are among the most sensitive and specific [25,26]. The discovery of PCR in the1980s offered scientists of a multitude of different disciplines an unprecedented opportunity to selectively amplify unique DNA or RNA sequences [27]. This revolutionary approach, enriched and refined by a variety of subsequent implementations, has been successfully employed to investigate basic biologic questions in plant pathology. Important breakthroughs have been made in the study of the genetics, ecology, and epidemiology of important plant pathogens, from the exploration of pathogens’ population structures to investigations into airborne inoculum dispersal, fungal mating-type analyses, fungicide resistance examinations, etc. Major advances have been made in plant disease diagnosis, with the unparalleled possibility of detecting plant pathogens from minute amounts of DNA—even from asymptomatic tissue and from a variety of matrices [28]. The application of innovative molecular diagnostic techniques provided a remarkable impetus for surveillance and monitoring campaigns for the prevention of pathogen introductions into disease-free areas [25,29,30]. From this point on, researchers’ efforts have been concentrated on achieving the rapidity, simplicity, and cost-effectiveness of detection tools without losing sensitivity, specificity, and accuracy features [19].

## 2. The Point-of-Care (POC) Diagnosis of Plant Diseases

A step forward in modern diagnostics is evident in the development of rapid and cost-effective methods to be applied directly at the point of care (POC) [31,32]. Indeed, moving testing closer to the site of sampling (in the case of plant pathogens, in the field) has been recognized as a significant advantage in terms of controlling and managing diseases, making it possible to reduce the delay between sampling and pathogen identification and, as a consequence, making the application of control measures more effective [33]. Even if PCR-based testing methods are highly sensitive and specific, they require a laboratory with the necessary facilities. Although a number of companies have produced real-time PCR and mobile PCR platforms for use in field detection [29,34], this equipment is often bulky and expensive, making the whole system sophisticated and unsuited for field testing. The main reason is that PCR-based reactions need temperature cycling to work properly, often coupled with equipment with controllers and sensors able to record the minimal variations of the fluorescence signal during amplification. On miniaturized thermal cyclers that can be used in the field, precise temperature control is still difficult, introducing challenges for the correct deployment of PCR reactions [35]. In addition, reactions take a long time (about 2 h) to be prepared and carried out due to the high quantity of reagents necessary and need to be performed with purified samples, usually requiring complex DNA or RNA extraction protocols and expert operators to be implemented. In order for field deployment to be possible, a POC diagnostic method should be suitable for applications in extreme field conditions, maintaining high levels of sensitivity and specificity, in addition to being robust and rapid and having user-friendly equipment (simple to perform and interpret in a few steps with minimal training) [17,33]. Among alternative rapid and user-friendly methods developed for on-site detection of plant pathogens, immunoassay-based tools (e.g., lateral flow device strips) are considered a valuable solution [36]. Although the development of antibodies for plant viruses has usually been quite successful, immune-based approaches are lass suitable and accurate and usually more expensive for more complex organisms such as fungi [26]. As a consequence, immunoassays may not be sufficiently sensitive or specific to identify pathogens at the required taxonomic level, as is often required for a quarantine species [35]. A promising and emerging alternative for POC detection is the use of molecular tools based on isothermal amplification of DNA or RNA sequences [32,37]. Among these, Loop-mediated isothermal AMPlification (LAMP) [38] is being increasingly employed in plant disease diagnostics due to its versatility and the wide possibilities it offers at the POC [39] (Table 1).

## 3. The LAMP Technique for POC Application

LAMP is a recently developed reaction [38] that can amplify a few copies of a target genomic sequence to 10^9^ in less than an hour under isothermal conditions. It relies on auto-cycling strand displacement DNA synthesis, which is performed by a DNA polymerase with high strand displacement activity. Usually, the method makes use of the large fragment of the Bst DNA polymerase obtained by *Geobacillus stearothermophilus*, which is fused to the maltose binding protein (MBP) of *E. coli*. MBP is used for purification and remotion of the fused proteins by cleavage, while the large fragment of the Bst DNA polymerase, containing the 5′–>3′ polymerase activity but lacking the 5′–>3′ exonuclease activity, is used in the reaction to amplify and displace DNA [183]. On the contrary, RNA targets require an additional reverse transcriptase enzyme that can be simultaneously added to the amplification reaction [184]. Since at the temperature required for LAMP functioning, double-strand DNA is in dynamic equilibrium, the technique does not require a step of heat denaturation to start amplification [37]. As in the original reaction, a set of two specially designed inner primers [forward inner primer (FIP) and backward inner primer (BIP)] and two outer primers (F3 and B3) that can hybridize six different regions of the target genomic sequence are strictly necessary for LAMP functioning [38]. A third pair of primers (backward and forward loop primers) hybridizing to the stem loops, except for the loops that are hybridized by the inner primers, can be added optionally to the reaction to accelerate target amplification [182]. The amplification of genomic sequences by the LAMP reaction starts with the conversion of a template in a dumbbell-shaped single-strand DNA/RNA that is used as starting material to initiate the cycling phase of the reaction. At the end of this step, DNA/RNA products that are connected to an inverted repeat structure in the amplified region are produced. All the products produced in the second phase then serve as templates for a series of elongation and recycling phases that generate amplified DNA/RNA products of various stem lengths and allow for an exponential amplification of the target sequence [38,184]. Since its first implementation, LAMP has been widely applied in health screenings of both animals and plant diseases, as well as in food safety testing, genetic testing, and analysis of environmental samples [185]. Among molecularly based diagnostic methods, this technique is widely preferred for point-of-care application, as it guarantees a rapid, simple, accurate, and cost-effective diagnosis [186].

### 3.1. Decisive Breakthroughs: Isothermal Functioning and User-Friendly Features

Among the features that make the LAMP technique competitive in field applications is its isothermal functioning. LAMP can amplify DNA at a constant temperature of 65 °C [38]; many authors have used this temperature to optimize and apply protocols based on this technique to detect and analyze plant pathogens [17]. Although many authors have also explored the effects of changing the LAMP amplification temperature (60–64 °C) [168], the isothermal functioning of the reaction provides several advantages for field suitability with respect to PCR-based methods [35]. As it does not require thermal cycling, there is no need for precise and bulky instrumentation to use LAMP and monitor its products, lowering the complexity of the required equipment [37]. As a consequence, many platforms and devices have been developed and are now on the market, able to meet the requirements of end users [187]. Although some authors have applied LAMP on suitable laboratory equipment (e.g., a real-time thermocycler), tools to be applied into the field have been widely developed and are constantly researched in order to find improvements able to provide the most effective route for routine use (e.g., heated block, portable instruments that can read amplification fluorescence, smartphone-based devices, and microchips) [185,188]. The application of the LAMP method is further simplified by the small quantity of reagents needed for reactions in comparison to PCR-based molecular detection tools. In addition, PCR reagents are usually very sensitive to temperature, requiring multiple steps to correctly prepare a reaction, as well as training and experience to reproduce it effectively. On the contrary, LAMP reagents are highly thermostable, allowing for lyophilization and storage at room temperature for up to several months [32].

### 3.2. Rapidity and Cost Effectiveness

Apart from the advantages with respect to user-friendly requirements, all the described features provide benefits in terms of the rapidity and cost of the LAMP technique. As an example, Panno et al. [186] calculated that, considering an amplification analysis without including a result visualization phase, a conventional end-point PCR costs at least USD 12, while the cost of LAMP is about USD 3, making this test very economical and, hence, widely usable. Another advantage of LAMP not to be ignored is that it helps to save time, as it is an efficient reaction capable of reaching the exponential phase of amplification in minutes, speeding up pathogen detection and subsequent management [16]. Indeed, unlike other nucleic acid amplification methods (e.g., PCR), LAMP results can be obtained in very little time (30–60 min), allowing for immediate diagnosis [157,168]. However, although LAMP is, in general reported, as less time-consuming than PCR-based methods, it must be considered that its rapidity and performance, as is the case for all nucleic acid-based detection methods, are linked to several factors that need to be analyzed during assay optimization [19]. Among the features that can influence the rapidity of the LAMP reaction, the type of analyzed tissues must be considered, as well as the genes chosen as targets and the volume and reaction characteristics (see Section 3 and Section 4).

### 3.3. Using Unprocessed DNA Samples

Another feature that can influence the rapidity and time needed for a reaction is the preparation of DNA samples, mainly the extraction and purification phases. When based on PCR methods, an effective diagnosis of plant pathogens—in particular, when dealing with fungi—requires efficient methods of genomic extraction that need to be capable of lysing plant cells (e.g., leaves, stems, wood, etc.), recovering a quantity of DNA/RNA sufficiently high and pure to be amplified without repercussions for assay performance [17]. Such protocols usually rely on the use of commercial extraction kits, requiring lab supplies to be applied (e.g., centrifuges, heating blocks, chemicals, incubation ice, etc.), making them unsuitable for point-of-care analysis [26,43,44,55,62,72,95,104]. Although suitable field methods for genomic material extraction have been widely developed and researched, the resulting extraction purity and yields are often not comparable to those obtained in the lab, especially when dealing with hard matrices (e.g., woody tissue) [189,190]. This could compromise PCR results, in addition to generating false negatives [191]. On the contrary, LAMP-based methods are usually reported as more tolerant to inhibitors than PCR, as the used enzymes are assessed as more robust [192]. As an example, Kaneko et al. [193] compared the inhibitor reaction tolerance and performance of LAMP and PCR by testing unextracted samples retrieved from different culture media or biological substances. They found that the tolerance of LAMP to inhibitors was higher than that of PCR, suggesting that DNA extraction can be omitted when dealing with this technique. Although a higher tolerance of the LAMP reaction to inhibitors has been reported for many compounds and biological substances, Nixon et al. [194] described matrices that can inhibit LAMP to a greater extent than PCR. This demonstrates that particular matrices can exhibit different levels of inhibition on different nucleic acid amplification methods. Hence, researchers should confirm the absence of inhibition for each potential compound when describing suitable POC extraction methods. Apart from this, many authors have described the possibility of applying LAMP coupled with relatively impure sample materials as templates [192]. This result was explained, in part, by Nwe et al. [191], who found that the presence of inhibitors during the LAMP reaction progress can delay the onset of amplicon formation and quencher fluorescence without usually affecting the end-point measurement of LAMP amplicons. Methods of crude extraction that can be implemented such as boiling, mincing, or a mixture of both steps, have been used with LAMP to further simplify the DNA extraction phase, avoiding laborious sample preparation before analysis [57,59,61,67,70,81,82,100,107,118,124,126,127,134,149,150,153,155,157,164,171,177,179,181]. These methods have been applied for the detection of plant pathogens from different plant matrices, demonstrating the capability of LAMP to return positive detection results without the need for DNA purification [157,168]. Even if detection is successful when amplifying samples processed with crude extraction, a decrease in assay performance was observed, mainly affecting the capability of LAMP to detect low quantities of inoculum. In addition, the method is difficult to replicate, affecting the reproducibility and consistency of the protocol [17]. Although crude extraction methods may not be suitable where early detection is concerned, further research is needed to evaluate their performance, as their simplicity and intuitiveness make them promising for various POC applications (Table 2).

## 4. Detection, Monitoring, and Quantification of LAMP Products

In theory, the requirements of a POC diagnostic method should accomplish the criteria summarized for the first time in 2003 by the World Health Organization (WHO) in the ASSURED guidelines [17,37]. As the acronym ASSURED states, the features identified for a POC method are Affordable, Sensitive, Specific, User-friendly, Rapid and robust, Equipment-free, and Deliverable to end-users, recognizing accuracy, accessibility, and affordability as the three most important keys and drawing attention to real-time connectivity and ease of specimen collection, which have acquired importance in recent years [195]. Although the LAMP technique has been described as a promising technique in terms of ease of use, cost-effectiveness, and lack of equipment (see Section 2), its detection performance is closely related to the optimization and setup of each analysis method [17,19]. The optimization of a molecularly based assay is, indeed, a necessary process that allows for the analysis of the protocol performance parameters (e.g., analytical specificity, analytical sensitivity, selectivity, reproducibility, and repeatability) used to acquire values as near as possible to those resembling what is ideally required by its supposed use [28]. Hence, as a preliminary step, it is necessary to define the protocol based on its intended application. Then, assay validation can be carried out by defining testing volumes, feasibility, diagnostics yields, and any other criteria identified as useful for achieving the best performance. Among the reported criteria, sensitivity, specificity, and the method used to monitor and visualize results are the most important parameters to verify the POC suitability of each tool.

### 4.1. Specificity and Sensitivity

The ideal POC detection tool in terms of specificity and sensitivity is one with parameters that get closer to those of laboratory-based diagnostic protocols as much as possible. Specificity is described as the capability of an assay to detect the target gene chosen for the organism to be diagnosed without returning false positives [196]. Since the LAMP reaction works with four or six primers targeting six or eight different genomic regions, it is usually reported as a highly discriminating and capable technique [184]. Indeed, many authors have reported high analytical specificity values (95–100%) for optimized LAMP-based assays, usually surpassing or comparable to those obtainable by PCR-based protocols [197]. Despite the potential of LAMP concerning specificity, it must be considered that the success of molecularly based identification is closely linked to the primer design step [198]. Indeed, during this phase, the genomic region to be selected as an amplicon needs to be accurately analyzed concerning intraspecific and interspecific genetic variation in order to choose an appropriate genomic marker able to identify the target with certainty, avoiding overlap with different organisms [19]. Hence, even LAMP-based assays could return lower specificity than PCR if the genomic region selected as a target cannot assure a high level of discrimination between the target and other genetically related organisms. In addition, some species could undergo genetic rearrangement that could influence the specificity of previously developed assays, invalidating their performance [51,199]. The specificity of a developed assay could also be influenced over time by the emergence of new haplotypes in the population when dealing with organisms able to undergo genetic recombination during reproduction or by the mutation of target genomic regions due to the possibility of insertions, deletions, or substitutions of single or multiple nucleotides (e.g., migration or genetic drift) [9]. Hence, specificity reconfirmation is a good practice in molecularly based detection before application for surveillance purposes. Although a lower specificity can be tolerated, if the harm of overtreatment is much less critical than missing the diagnosis of an infection, the use of a single genomic marker could, in some cases, not be sufficient to definitely diagnose the searched pathogen [19,195]. This is particularly true for fungi, for which conflicts in the definition of different taxonomic entities have been raised by the advent of DNA-based identification, often requiring multi-locus diagnostic testing to properly identify a pathogen at the species level [200]. The other parameter that can affect the performance of a LAMP assay is sensitivity, which is defined as the minimum amount of a target that can be detected by the protocol in a sample [196]. Since it is strictly connected to the minimum concentration of DNA or RNA that the assay is able to detect, this parameter can be linked to the ability of the protocol to return or avoid false-negative results and is related to its ability to diagnose the pathogen at low inoculum rates (e.g., asymptomatic or latent phases) [16]. Usually, LAMP is reported as capable of returning high sensitivity values, reaching target quantities as low as six DNA copies. This trend has also been confirmed by numerous studies comparing PCR and LAMP-based detection, in which LAMP sensitivity has been assessed to be 10 to 100 times higher than that of PCR, reaching values from 87% to 100% depending on the protocol features [197]. However, some authors have also reported lower sensitivity for LAMP reactions when compared to PCR or qPCR detection methods, frequently when dealing with the optimization of LAMP assays targeting plant pathogens in environmental samples (e.g., plant tissues and soil) [168]. This could be partially due to the low purity of environmental samples that, coupled with carried contaminants, could directly affect the amplification reaction and, as a consequence, the detection performance (e.g., sensitivity, accuracy) [191]. On the contrary, LAMP sensitivity seems not to be affected by the presence of non-target DNA amounts in the analyzed samples [157]. However, the nature of the target gene can have an influence on the detection sensitivity. It has been reported that selecting a target with a high copy number in the genome can increase the sensitivity of the assay, so multicopy genes or non-coding gene regions are usually preferred over single-copy regions [28]. Even so, among the multicopy genes or non-coding gene regions widely used as the “barcode” reference sequence, e.g., the internal transcribed spacer (ITS) region, there are generally highly conserved traits, which, despite presenting a high degree of variation between closely related species, may not be specific enough for discriminating some organisms (e.g., *Fusarium* spp.). Therefore, during the optimization and validation of each protocol, it is necessary to strike a compromise between the best achievable performance in terms of sensitivity and specificity. Alternatively, methods to increase the specificity and sensitivity of an assay can be searched for. Apart from reaction optimization, authors have reported the possibility of obtaining higher LAMP sensitivity by adding an initial denaturation/melting step at 95 °C to the amplification reaction [37], by using the amplicons with several inverted repeats produced by amplification to increase the sensitivity of LAMP hybridization assays (e.g., coupled with ELISA or lateral flow strips), or by the application of a hybridization probe-based protocol [31]. Other approaches concentrate on understanding the causes of primer–dimer pairs or nonspecific amplification production by LAMP reactions in an attempt to identify their influence on sensitivity and specificity, researching ways to limit their impact on protocol performances [201]. In addition, the use of different enzymes and their effects on LAMP amplification performance have been investigated [202].

### 4.2. Visualization and Quantification of Reaction Products

The achievement of sensitivity, specificity, accuracy, stability, and simplicity for LAMP reactions depends not only on the performance of DNA/RNA amplification but also on the method chosen for monitoring reaction products. LAMP products can be visualized using both indirect and direct methods [203].

#### 4.2.1. Indirect Methods

**Figure 1:**
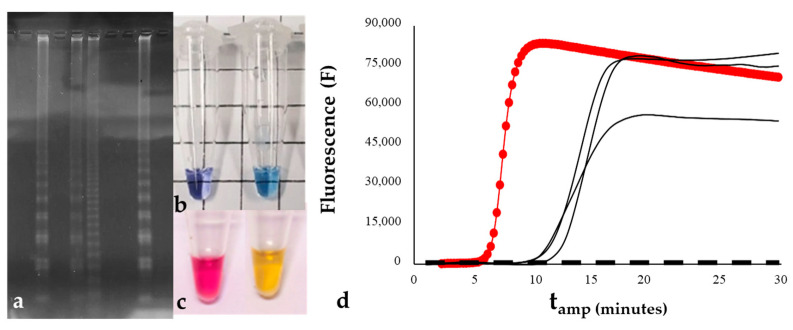
Indirect and direct methods that can be used for LAMP product visualization: (**a**) gel electrophoresis; (**b**,**c**) negative and positive controls of a LAMP reaction monitored with colorimetric dyes; (**d**) LAMP amplification curves obtained by real-time analysis of the fluorescence emitted by the reaction. Curves can be displayed directly on the instrument monitor.

Indirect methods include all those requiring post-amplification steps to be implemented. Apart from gel electrophoresis (Figure 1a), which needs a lab to be applied, since they do not usually return consistent results, the most common indirect methods used for LAMP reactions are turbidity and hydroxy naphthol blue (HBN)- or calcein-based methods [204]. The functioning of turbidity is usually based on the insertion of deoxynucleotide triphosphates (dNTPs) into the reaction mixture. This compound is incorporated in DNA strand polymerization, releasing pyrophosphate ions that, when in high concentration, can react with bivalent metal ions present in the buffer (e.g., magnesium, calcium, and manganese), precipitating as pellets [86,93,96,111,114,120,122,123,124,155]. When coupled with optical instruments (e.g., turbidimeters or spectrophotometers), it can be used to quantify the number of copies of the target gene in real time [205]. However, the positivity of reactions can also be assessed by observing the tubes with the naked eye after amplification (Figure 1b,c). Even if there is no further instrumental cost nor post-amplification contamination risk in this monitoring method, the stability of samples is achieved only for a short time after the amplification. In addition, the subjectivity associated with the interpretation of results, coupled with a high detection limit, makes it possible to overcome detection mistakes. Alternatively, colorimetric indicators, e.g., hydroxy naphthol blue (HBN) and calcein (Figure 1b,c), can be employed in LAMP monitoring. HBN develops a violet color as a consequence of its binding to pyrophosphate produced during amplification, which causes a decrease in the concentration of Mg^2+^ [206]. Similarly, calcein can quench manganese ions before amplification. When the reaction proceeds, manganese binds to newly formed pyrophosphate, releasing calcein to complex free magnesium and recovering bright-green fluorescence [207]. Hence, the positivity of a sample can be assessed visually by observing the post-amplification color change [41,50,53,58,61,63,66,68,70,75,76,77,84,87,88,93,94,98,99,111,119,124,125,127,128,134,141,149,161,177]. Although color-based assessment of sample positivity may be particularly suitable for field use, observing color changes in different light environments or at different times of the day could lead to false negatives or sensitivity losses [31]. In addition, the necessity of opening the tubes to add dyes in post amplification makes the method extremely vulnerable to carryover contamination, and it has been reported that some colorimetric dyes can have inhibitory effects on the LAMP reaction.

#### 4.2.2. Direct Methods

Another possibility is the use of intercalating dyes (e.g., SYBR Green I or EvaGreen) as direct a detection method that does not require post-amplification steps [46,49,51,54,55,65,85,95,102,104] (Figure 1d). As fluorescence signals are emitted when the dyes bind to double-stranded DNA (dsDNA), this method permits the real-time monitoring of the reaction [157]. It has been widely coupled with field-deployable fluorometers (e.g., Genie^®^ instruments), and for this reason, it is particularly well-suited for POC detection, making it possible to simplify interpretation of results, which are usually displayed as curves on device monitors [1,45,52,56,69,77,78,81,107,112,118,126,133,150,153,157,159,162,164,168,171,175,179,181]. In addition, using this method, LAMP products can be quantified by extrapolating the initial concentration of a sample from the calibration standards, usually referred to as the amplified product, and the detection threshold, which, in LAMP, is defined as the time at which a positive fluorescent signal is recorded [79,157]. The major drawback of this fluorescence-based monitoring method is that intercalating dyes react with dsDNA as a form of nonspecific quenching, meaning that every time something is amplified, a positive result is obtained. As a consequence, the rate of false positives could increase, leading to a decrease in diagnostic specificity. Moreover, the fluorescence of intercalating dyes is usually emitted in a single wavelength, not allowing for the simultaneous detection of two or more target sequences in the same sample, as multiplex methods allow.

#### 4.2.3. Probe-Based Methods

Attempts to solve these issues while maintaining the simplicity of real-time monitoring have been made with the implementation of probe-based methods for LAMP with single or multiple labels [48,51,56,57,69,74,79,100,163,167,169]. Many probe-based LAMP monitoring methods are available; their different typologies and mechanisms were discussed and reviewed by Becherer et al. [187]. Most of them work with a dye-labeled primer incorporated into a target-specific amplicon in the LAMP reaction. Most probe-based methods have been applied with LAMP, mainly for the detection of viruses and bacteria (e.g., *Ralstonia solanacearum* and *Salmonella enterica*), but few LAMP methods targeting fungal and pseudofungal plant pathogens have been developed (e.g., *Magnaporthe oryzae* and *Phytophthora infestans*) and many of these rely on the technology implemented by Kubota et al. [208]. This method relies on assimilating probes composed of two labeled strands. The first strand contains a fluorescent dye at the 3′ end and is built on a loop primer that is incorporated in its sequence. The second one is the complementary strand with a dark quencher at the 5′ end. Once the loop primer is amplified, the two strands separate, resulting in the destruction of the probe and allowing the emission of fluorescence. Hence, its correct functioning requires the specific amplification of the included loop primer. As loops are usually shorter than LAMP amplicons and amplification fluorescence is strictly related to the selected loop, this method can be used to increase the specificity of an assay, making it possible to select the genomic part of the target to which the fluorescence signal is linked. As an example, Stehlíková et al. [56] assessed the ability of this method to target *F. circinatum* with higher specificity than a conventional LAMP reaction built with the same primers and targeting the same genetic region. However, in the same paper, a loss of sensitivity was assessed when applying LAMP with the developed assimilating probe. Many new methods to ameliorate LAMP specificity and sensitivity with probe technology have been investigated in recent years [203]. As an example, Cui et al. [48] incorporated a TaqMAN probe in a LAMP reaction to specifically detect *Colletotrichum siamense* in soil samples. The authors obtained higher specificity than the conventional LAMP reaction used for comparison, without losses in sensitivity. The values of the limit of detection obtained by these authors using a TaqMAN probe in LAMP reaction were higher than those achieved by qPCR techniques previously developed to target the same pathogen, making TaqMAN-LAMP technology a promising alternative to improve LAMP applications. As probe-based methods allow for marking with dyes emitting in different wavelengths, they make it possible to perform multiplexing on LAMP reactions [209]. The possibility of simultaneously amplifying and monitoring multiple sequences in a single analysis could further improve the suitability of LAMP for POC applications, reducing the required time, in addition to being cost-effective and requiring a minimum amount of starting samples [91,119,156,210]. However, it has been reported that the complexity of the LAMP reaction could affect the performance of such assays, decreasing sensitivity and specificity when combining more primer sets and DNA targets in the same analysis, particularly concerning complex organisms such as fungi [203]. Hence, probe-based multiplex applications of LAMP reactions are quite undeveloped, mostly concerning viruses and bacteria [10] applied as parallelized single reactions that do not require the mixing of several sets of primers [209].

#### 4.2.4. Lab-on-a-Chip (LOC) Applications

**Figure 2:**
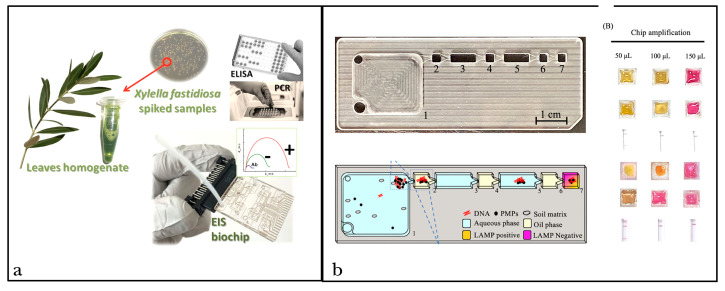
Examples of lab-on-a-chip tools developed to detect plant pathogens. (**a**) LOC technology based on PCR reaction (credit: Chiriacò et al. [212]) (**b**) LOC device based on LAMP reaction. Positive results are visualized as yellow, and negative results are represented as pink (credit: Changtor et al. [40]).

To further simplify the process of result monitoring and visualization, in recent years, the possibility of applying lab-on-a-chip (LOC) technologies (Figure 2) able to integrate the entire diagnostic process on simple miniaturized platforms as microchips, paper-based microfluidics, or smartphone-based devices has attracted attention to POC diagnostics [185]. Several microchip- and paper-based platforms have been developed recently for LAMP amplicon detection coupled with each previously described monitoring method [188] (Figure 2a). Although novel monitoring technologies that can further simplify LOC processes are researched constantly (e.g., giant magnetoresistance, bioluminescence, and pH sensing), most currently available LOC-based methods for LAMP product detection rely on optical electrical and electrochemically based monitoring methods [40,74,169] (Figure 2b). Electrochemical functioning is usually based on redox molecules intercalated to amplicons and has been coupled with these technologies for both end-point and real-time monitoring. This approach is generally reported as highly sensitive and capable of providing an accurate test without access to laboratories. On the contrary, the optical method of visualization of products is based on turbidity analyses, with the disadvantages previously described for this method. Although these techniques have been thoroughly developed in medical diagnostics, the detection of plant pathogens based on these tools is still limited, and LOC-based assays to target plant pathogens have been mainly developed in combination with PCR reactions [211,212]. As LOC technologies have the potential to work without the necessity of a lab on handheld, portable, low-cost tools without requiring skills to be applied, they are promising as POC methods, allowing for their use in resource-limited settings [213,214].

#### 4.2.5. Methods Coupled with CRISPR-Cas Technology

A step forward in developing LAMP assays for low-resource settings was made in recent years, given the possibility of coupling the LAMP reaction with regularly interspaced short palindromic repeats (CRISPR)-associated (Cas) technology. This technology, retrieved from microbic adaptive immune defense systems against invasion by extraneous genetic elements, is based on the activation of Cas protein to provide nonspecific cleavage of single-stranded DNA (ssDNA). As a result, it has been demonstrated as a useful technique to develop diagnostic tools with enhanced specificity and sensitivity. As an example, Li et al. [74] developed a LAMP assay coupled with CRISPR-Cas12a to specifically detect *Fusarium temperatum*. The reaction, which is highly specific and sensitive, can be implemented in a single Eppendorf tube, providing results as a colorimetric color change that is easy to interpret. Hence, although the technique is considered to be in its first stage of implementation, it has great potential to revolutionize the developments of POC diagnostic methods.

## 5. Drawbacks of LAMP-Based POC Methods

Although LAMP seems to be a promising technique for POC detection capable of maintaining high sensitivity and specificity without exceeding analysis costs on simplified devices, it is not a perfect technique, with some drawbacks and limitations. First, the functioning of the LAMP reaction is complicated and unintuitive, requiring genetics and diagnostics skills to be correctly set up [38,184]. This is reflected in the difficulty of designing primers that can achieve good performances in terms of sensibility and sensitivity, as a POC method for early detection requires [39,79]. Although software for the design of primers suitable for LAMP reactions have been developed, not all such software is freely available, limiting access to the technique for non-lab users [31]. In addition, free software for LAMP primers design is usually simpler in terms of available analyses, meaning that even if primers can be designed, their correct functioning is not guaranteed. Although the difficulty of LAMP design and optimization affects more researchers than end users, the required skills can limit both affordability and the application of LAMP by non-specialized personnel [203]. Further limitations for non-specialized applications are associated with the materials (e.g., reagents) needed for LAMP execution, which can be of restricted availability in some countries [39], as well as the necessity of high-performance liquid chromatography (HPLC) purification for the two longest primers [198]. Furthermore, a LAMP-based POC assay integrating the entire process from sample preparation to visualization of results is still elusive. This may be partly due to the state of infancy of the technologies, which although simplified and more cost-effective than PCR instruments, are still expensive and not entirely suitable for applications under extreme field conditions (e.g., when exposed to adverse weather) [185]. Indeed, the field application of LAMP diagnosis is still limited, only occasionally including an in-field validation in LAMP optimization works concerning plant pathogen detection. Different conditions and users could influence protocol performance, requiring on-site validation of all the protocol steps before application [17,19]. A suitable field extraction protocol is often not included in assay optimization, with sampling and sample storage among the steps that can invalidate the results of a LAMP diagnosis [196]. This is especially true for plant pathogen detection, which is rarely discussed when describing a new LAMP protocol. Indeed, false negatives can be obtained if the applied sampling method is not systematic and statistically consistent, as the diagnostic protocol might not be able to detect asymptomatic or low-inoculum infections, which is also relevant when applied to different plant tissues. Hence, the performance of such protocols (e.g., sensitivity) could be underestimated. In addition, long storage of collected samples could lead to the invasion of tissues by secondary organisms, leading to a decline in the target population, affecting the results. Since results are usually validated through comparison with the reference DNA samples included in each test, another important factor is the proper storage of the DNA, which is necessary to prevent degradation and ensure its stability [196]. Hence, although progresses has been made in the direct use of LAMP on site, the main limitation is that it still requires a lab base to be implemented [198].

## 6. Conclusions and Future Perspectives

**Figure 3:**
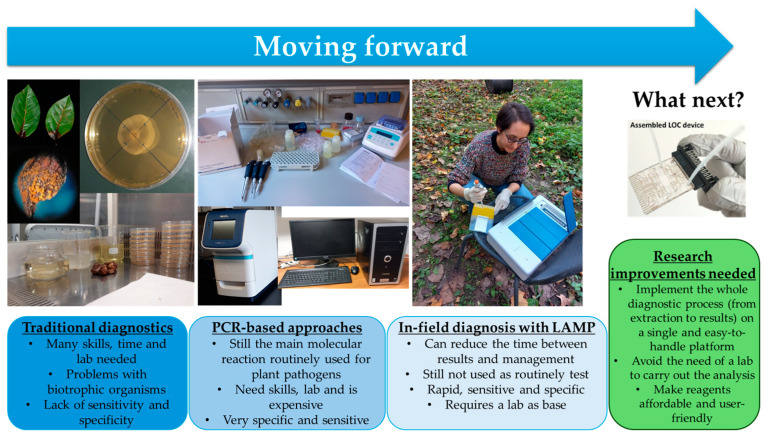
Diagrammatic representation of the evolution of diagnostics methodologies for IAPP detection and future perspectives. Pictures of the LOC device were obtained from Chiriacò et al. [212]. The LAMP instrument in the picture is developed and marketed by Enbiotech s.r.l.

The advent of nucleic acid-based approaches for plant pathogen detection has revolutionized plant pathology, offering the possibility of obtaining accurate identifications of pathogens without the necessity of plant pathology (mycology, bacteriology, and virology) skills, as the study of morphology and symptomatology requires [29]. Since the development of PCR [27], molecular-based techniques have been applied in plant pathology for a wide range of uses, ranging from monitoring issues to research questions [25,26,30]. As they are capable of being highly specific and sensitive, such techniques have attracted increased interest as surveillance methods able to detect pathogens in asymptomatic or latent phases to prevent new introductions, as well as to limit their uncontrolled spread in new environments once introduced [16]. Over the years, the importance of acquiring rapid responses (early diagnosis) has come to assume a key role because the prompt implementation of control measures depends on the speed with which a causal agent is identified [18,33]. Hence, the possibility of directly applying these kinds of techniques on site has been investigated, and new simplified and cost-effective molecular technologies have been developed [17,19,31]. Among these, LAMP has demonstrated the greatest potential as a POC method, offering fast, sensitive, specific, robust, and cost-effective analysis that can be implemented on easily handled portable devices [35]. In the decade following its first publication [38], LAMP has been used for plant pathogen detection in more than 250 peer-reviewed research articles. Since 2012, the number of LAMP-based papers has increased exponentially, including those reporting on the identification of the source of infection for plants, animals, and humans, with simpler platforms developed to allow for the use of molecular techniques by non-specialized personnel [39]. Apart from research, the number of commercially available devices and LAMP-based kits has also increased in an attempt to bridge the gaps in molecular diagnostics, enabling non-lab users (e.g., farmers) to apply them. Concerning damaging pathogens and pest surveillance, the application and wide distribution of molecular POC methods could improve the prevention and control of IAPP invasion, limiting ecological damage and the costs of managing new disease outbreaks [1,2,17,19]. However, these methods are still not the routine application choice when dealing with field screening or analysis of imported plants, fruits, and vegetables, probably due to technical difficulties that cannot yet be overcome (Figure 3). Although with the implementation of LAMP, molecularly based detection has come close to being within everyone’s reach, there are many challenges that still need to be solved to realize its effective use as a POC method. These challenges include (1) the implementation of effective and reliable methods for on-site DNA/RNA extraction and purification; (2) the availability of materials for reaction implementation, ranging from primers to necessary platforms, which must be readily available for use a low cost; and (3) the impossibility of applying LAMP without having a lab as a base. Hence, further research is still needed to fully exploit the potential of this tool, enabling non-skilled people to apply it.

## Figures and Tables

**Table 1:** List of LAMP protocols developed to target specific plant pathogens (fungi, oomycetes, viruses, bacteria, phytoplasmas and nematodes) and insects. For each protocol, the following features are highlighted: target samples used for diagnosis in plant tissue/insects, type of LAMP reaction, method used for the visualization of amplicons, and method of DNA/RNA extraction.

Disease Name	Targeted Samples	Kind of LAMP Reaction	Method of Product Visualization	DNA/RNA Extraction Method	Reference
FUNGI AND OOMYCETES					
*Agroathelia rolfsii*	Soil	Labeled primers for chip-based reaction	Colorimetric dyes	Immiscible filtration assisted by surface tension (IFAST)-based; chip-based	Changtor et al. [40]
*Botrytis cinerea*	Fruits and flowers	Conventional ^1^	Real-time fluorescence; HBN ^4^	CTAB ^6^-based; lab kit	Tomlinson et al. [41]; Duan et al. [42]
*Bremia lactucae*	*Lactuca sativa*	Conventional ^1^	Real-time fluorescence	Lab kit	Farmer et al. [43]
*Calonectria henricotiae* ^(IAPP)^	*Buxus* spp.	Conventional based on OmniAmp^TM^ RNA & DNA LAMP kit (Lucigen Corporation, Middleton, WI, USA)	Digital gel electrophoresis	Lab kit	Malapi-Wight et al. [44]
*Calonectria ilicicola*	*Persea americana*	Conventional ^1^	Real-time fluorescence on a portable instrument	Lab kit	Parkinson et al. [45]
*Calonectria pseudonaviculata* ^(IAPP)^	*Buxus* spp.	Conventional based on OmniAmp^TM^ RNA & DNA LAMP kit (Lucigen Corporation, Middleton, WI, USA)	Digital gel electrophoresis	Lab kit	Malapi-Wight et al. [44]
*Ceratocystis platani* ^(IAPP)^	*Platanus* spp.	Conventional ^1^	Real-time fluorescence on a portable instrument	Lab kit and field DNA extraction kit (OptiGene)	Aglietti et al. [1]
*Colletotrichum falcatum*	*Saccharum officinarum*	Conventional ^1^	SYBR Green I dye	CTAB ^6^-based	Chandra et al. [46]
*Colletotrichum gloeosporioides*	*Fragaria × ananassa*	Conventional ^1^	HBN ^4^	Lab kit	Wu et al. [47]
*Colletotrichum siamense*	Soil samples and *Fragaria* spp.	Conventional ^1^ and TaqMAN-based	Gel electrophoresis, red dyes, and real-time fluorescence	CTAB ^6^-based and lab kit	Cui et al. [48]
*Coniella granati*	*Punica granatum*	Conventional ^1^	SYBR Green I and gel electrophoresis	Lab kit	Yang et al. [49]
*Cronartium ribicola* ^(IAPP)^	*Pinus* spp.	Conventional ^1^	Colorimetric dyes	CTAB ^6^-based and field method	Kozhar et al. [50]
*Dactylonectria macrodidyma*	*Persea americana*	Conventional ^1^	Real-time fluorescence on a portable instrument	Lab kit	Parkinson et al. [45]
*Dothistroma septosporum* ^(IAPP)^	*Pinus* spp.	Based on assimilating probes; conventional	Real-time fluorescence; real-time fluorescence on a portable instrument	Lab kit	Aglietti et al. [51]; Myrholm et al. [52]
*Dothistroma pini* ^(IAPP)^	*Pinus* spp.	Based on assimilating probes	Real-time fluorescence	Lab kit	Aglietti et al. [51]
*Didymella bryoniae*	*Cucurbitaceae*	Conventional ^1^	Calcein	Lab kit	Yao et al. [53]
*Elsinoë necatrix* ^(IAPP)^	*Eucalyptus* spp.	Conventional ^1^	Real-time fluorescence	Lab kit and simple method	Van Heerden et al. [54]
*Fusarium acuminatum*	*Astragalus membranaceus*	Conventional ^1^	SYBR Green I and gel electrophoresis	Lab kit	Wang et al. [55]
*Fusarium circinatum* ^(IAPP)^	*Pinus* spp.	Conventional ^1^ and based on assimilating probes	Real-time fluorescence on a portable instrument; filter paper dipstick	Lab kit and crude method	Stehlíková et al. [56]; Meinecke et al. [57]
*Fusarium culmorum*	*Glycine max*	Conventional ^1^	HBN ^4^	CTAB ^6^-based	Zeng et al. [58]
*Fusarium fujikuroi*	*Oryza* spp.	Conventional ^1^	Gel electrophoresis	Lab kit and alkaline DNA extraction as a crude method	Ortega et al. [59]; Sanna et al. [60]
*Fusarium graminearum*	*Triticum* spp.	Conventional ^1^	Calcein and gel electrophoresis	Lab kit and crude method	Niessen et al. [61]
*Fusarium mangiferae*	*Mangifera* spp.	Conventional based on RAPD marker ^2^ sequence	Gel electrophoresis, SYBR Green, and real-time fluorescence	Lab kit	Pu et al. [62]
*Fusarium oxysporum* f. sp. *ciceris*	*Cicer arietinum*	Conventional ^1^	HBN and gel electrophoresis	CTAB ^6^-based	Ghosh et al. [63]
*Fusarium oxysporum* f. sp. *cubense*	*Musa* spp.	Conventional ^1^	Gel electrophoresis and SYBR Green I	CTAB ^6^-based	Li et al. [64]
*Fusarium oxysporum* f. sp. *cucumerinum*	*Cucumis sativus*	Conventional ^1^	Gel electrophoresis and SYBR Green I	Lab kit	Lan et al. [65]
*Fusarium oxysporum* f. sp. *fragariae*	*Fragaria × ananassa*	Conventional ^1^	Gel electrophoresis, HBN ^4^, and real-time fluorescence	Rapid method	Katoh et al. [66]
*Fusarium oxysporum* f. sp. *lactucae*	*Lactuca sativa*	Conventional ^1^	Real-time fluorescence	Lab kit and crude method	Franco Ortega et al. [67]
*Fusarium oxysporum* f. sp. *lycopersici*	*Solanum lycopersicum*	Conventional ^1^; based on universal QProbe	Visual dyes and real-time fluorescence on a portable instrument	Lab kit	Almasi et al. [68]; Ayukawa et al. [69]
*Fusarium oxysporum* f. sp. *melonis*	*Cucumis melo*	Conventional ^1^	Gel electrophoresis and HBN ^4^	Lab kit and crude method	Almasi et al. [70]
*Fusarium oxysporum* f. sp. *niveum*	*Citrullus lanatus*	Conventional ^1^	Gel electrophoresis, visual dyes, and real-time fluorescence	Lab kit	Peng et al. [71]
*Fusarium proliferatum*	*Zea mays*	Conventional ^1^	SYBR Green I and gel electrophoresis	Lab kit	Wang et al. [72]
*Fusarium solani*	*Astragalus membranaceus*	Conventional ^1^	SYBR Green I and gel electrophoresis	Lab kit	Wang et al. [55]
*Fusarium temperatum*	*Zea mays*	Conventional ^1^; nanoparticle probes coupled with CRISPR-CAS12a	SYBR Green I and gel electrophoresis; colorimetric dyes on a portable smartphone-based instrument	Lab kit	Shan et al. [73]; Li et al. [74]
*Fusarium tricinctum*	*Hordeum vulgare*; *Triticum* spp.	Conventional ^1^	Calcein	Lab kit and ultrasonication	Niessen et al. [75]
*Geosmithia morbida* ^(IAPP)^	*Juglans nigra*	Conventional ^1^	HNB ^4^ and real-time fluorescence	Lab kit	Rizzo et al. [76]
*Gnomoniopsis smithogilvyi* (syn. *Gnomoniopsis castaneae*)	*Castanea sativa*	Conventional ^1^	HNB ^4^ and real-time fluorescence on a portable instrument	Lab kit	Vettraino et al. [77]
*Hymenoscyphus fraxineus* ^(IAPP)^	*Fraxinus excelsior*	Conventional ^1^	Real-time fluorescence on a portable instrument	CTAB ^6^-based and suitable field method	Harrison et al. [78]
*Lecanosticta acicola* ^(IAPP)^	*Pinus* spp.	Based on assimilating probes	Real-time fluorescence	Lab kit	Aglietti et al. [51]
*Magnaporthe oryzae*	*Lolium perenne*; *Oryza* spp.	Based on assimilating probes; conventional ^1^	Real-time fluorescence and gel electrophoresis	Lab kit and alkaline DNA extraction as crude method	Villari et al. [79]; Ortega et al. [59]
*Marssonina coronaria* ^(IAPP)^	*Malus* spp.	Conventional ^1^	HBN ^4^ and gel electrophoresis	Rapid method	Ren et al. [80]
*Monilinia fructicola* ^(IAPP)^	*Malus* spp.; *Prunus persica*	Conventional ^1^	Real-time fluorescence on a portable instrument	Lab kit and crude method	Ortega et al. [81]; Poniatowska et al. [82]
*Monilinia fructigena* ^(IAPP)^	*Malus* spp.	Conventional ^1^	Real-time fluorescence	Lab kit and crude method	Poniatowska et al. [82]
*Monilinia laxa* ^(IAPP)^	*Malus* spp.; *Prunus persica*	Conventional ^1^	Real-time fluorescence on a portable instrument	Lab kit and crude method	Ortega et al. [81]
*Monilinia polystroma* ^(IAPP)^	*Malus* spp.	Conventional ^1^	Real-time fluorescence	Lab kit and crude method	Poniatowska et al. [82]
*Peronophythora litchii* ^(IAPP)^	*Litchi chinensis*	Conventional ^1^	SYBR Green I	Lab kit	Kong et al. [83]
*Peronospora destructor*	*Allium cepa*	Conventional ^1^	HBN ^4^ and gel electrophoresis	Lab kit	Yang et al. [84]
*Phytophthora cactorum* ^(IAPP)^	*Fragaria × ananassa*	Conventional ^1^	SYBR Green I, gel electrophoresis, and real-time fluorescence	Lab kit	Siegieda et al. [85]
*Phytophthora cambivora* ^(IAPP)^	None: applied only on axenic cultures	Conventional ^1^	SYBR Green I, gel electrophoresis, and turbidity	Lab kit	Li et al. [86]
*Phytophthora cinnamomi* ^(IAPP)^	*Carya cathayensis*	Conventional ^1^	HBN ^4^ and gel electrophoresis	Lab kit	Tong et al. [87]
*Phytophthora capsici* ^(IAPP)^	*Capsicum* spp.	Conventional ^1^	Calcein and gel electrophoresis	CTAB ^6^-based	Dong et al. [88]
*Phytophthora hibernalis* ^(IAPP)^	None: applied only on axenic cultures	Conventional ^1^	SYBR Green I, gel electrophoresis, and turbidity	Lab kit	Li et al. [86]
*Phytophthora infestans* ^(IAPP)^	*Solanum tuberosum*; *Solanum lycopersicum*	Conventional ^1^	SYBR Green I, HBN ^4^, gel electrophoresis, and LFD ^5^	CTAB ^6^-based and quick sodium hydroxide method; lab kit	Ristaino et al. [89]; Kong et al. [90]
*Phytophthora kernoviae* ^(IAPP)^	*Rhododendron* spp.	Conventional ^1^ and multiplex	Gel electrophoresis and LFD ^5^	CTAB ^6^ and LFD ^5^-based	Tomlinson et al. [91]
*Phytophthora nicotianae* ^(IAPP)^	*Nicotiana* spp.	Conventional ^1^	SYBR green I and gel electrophoresis	CTAB ^6^-based	Li et al. [92]
*Phytophthora ramorum* ^(IAPP)^	*Rhododendron* spp.; *Viburnum* spp.	Conventional ^1^ and multiplex	Gel electrophoresis and LFD ^5^; real-time fluorescence on a portable instrument	CTAB ^6^ and LFD ^5^-based; lab kit and field DNA extraction kit (OptiGene)	Aglietti et al. [1];Tomlinson et al. [91]
*Phytophthora sojae* ^(IAPP)^	*Glycine max*	Conventional; based on Loopamp DNA amplification kit (Eiken Chemical, Tokyo, Japan)	Optical density at 650 mm, HBN ^4^, and gel electrophoresis	Lab kit	Dai et al. [93]
*Phytophthora syringae* ^(IAPP)^	None: applied only on axenic cultures	Conventional ^1^	SYBR Green I, gel electrophoresis, and turbidity	Lab kit	Li et al. [86]
*Plasmopara viticola*	*Vitis vinifera*	Conventional ^1^	HBN ^4^ and gel electrophoresis	Rapid method	Kong et al. [94]
*Pyrenopeziza brassicae*	*Brassica napus*	Conventional ^1^	Real-time fluorescence	Lab kit	King et al. [95]
*Pythium helicoides*	*Euphorbia pulcherrima*	Conventional ^1^	Turbidity	Lab kit	Takahashi et al. [96]
*Phytopythium vexans*	*-*	Conventional ^1^	Real-time fluorescence and colorimetric dyes	Lab kit	Ghimire et al. [97]
*Puccinia triticina* ^(IAPP)^	*Triticum* spp.	Conventional ^1^	HBN ^4^, EtBr dye, and gel electrophoresis	Lab kit and CTAB ^6^-based	Manjunatha et al. [98]
*Puccinia melanocephala* ^(IAPP)^	*Saccharum hybrids* spp.	Conventional ^1^	Gel electrophoresis and colorimetric dyes	Lab kit and CTAB ^6^-based	Wu et al. [99]
*Raffaelea lauricola* ^(IAPP)^	*Persea borbonia*	Based on assimilating probes	Real-time fluorescence	Lab kit and crude method	Hamilton et al. [100]
*Rhizoctonia bataticola*	*Cicer arietinum*	Conventional ^1^	SYBR Green I and gel electrophoresis	Lab kit	Ghosh et al. [101]
*Talaromyces flavus*	*Fragaria × ananassa*	Conventional ^1^	Real-time fluorescence	Lab kit and quick method	Panek and Frąc. [102]
*Tilletia caries* ^(IAPP)^, *Tilletia laevis* ^(IAPP)^ and *Tilletia controversa* ^(IAPP)^	*Triticum* spp.	Conventional ^1^	EvaGreen (Biotium), gel electrophoresis, and real-time fluorescence	Lab kit	Pieczul et al. [103]
*Uromyces betae*	*Beta vulgaris*	Conventional ^1^	Real-time fluorescence	Lab kit	Kaczmarek et al. [104]
*Ustilago tritici* ^(IAPP)^	*Triticum* spp.	Conventional ^1^	Real-time fluorescence and gel electrophoresis	Based on phenol-chloroform-isoamyl alcohol	Yan et al. [105]
*Verticillium dahliae* ^(IAPP)^	*Olea europeae*	Conventional; based on RAPD ^2^ sequence	Visual dyes, gel electrophoresis, and real-time fluorescence on a portable instrument	Lab kit and crude method	Moradi et al. [106]; Megariti et al. [107]
VIRUSES					
Abaca bunchy top virus (ABTV)	*Musa textilis*	Conventional ^1^	GelRed and SYBR Green I	CTAB ^6^-based	Galvez et al. [108]
Apple chlorotic leaf spot virus (ACLSV)	*Prunus* spp.; *Malus* spp.; *Pyrus* spp.	RT-LAMP ^3^	SYBR Green I and gel electrophoresis	Lab protocol and CTAB ^6^-based	Peng et al. [109]; Lu et al. [110]
Apple stem pitting virus (ASPV)	*Pyrus* spp.	RT-LAMP ^3^	SYBR Green I and gel electrophoresis	CTAB ^6^-based	Lu et al. [110]
Banana bunchy top virus (BBTV)	*Musa textilis*	Conventional ^1^	Turbidity, SYBR Green I, GelRed, and gel electrophoresis	CTAB ^6^-based	Peng et al. [111]; Galvez et al. [108]
Cassava brown streak virus (CBSV)	*Manihot esculenta*	RT-LAMP ^3^	Real-time fluorescence on a portable instrument	CTAB ^6^-based	Tomlinson et al. [112]
Citrus leaf blotch virus (CLBV)	*Actinidia* spp.	RT-LAMP ^3^	SYBR Green I, gel electrophoresis, and LFD ^5^	CTAB ^6^-based	Peng et al. [113]
Citrus yellow mosaic badnavirus (CMBV)	*Citrus* spp.	Conventional ^1^	Turbidity, SYBR Green I, and gel electrophoresis	Lab protocol	Johnson et al. [114]
Citrus tristeza virus (CTV)	*Citrus* spp.	RT-LAMP ^3^	SYBR Green I and gel electrophoresis	Lab kit	Warghane et al. [115]
Coconut cadang-cadang viroid (CCCVd)	*Elaies guineensis*	RT-LAMP ^3^	Fluorescent dyes and gel electrophoresis	NETME method	Thanarajoo et al. [116]
Chrysanthemum chlorotic mottle viroid (CChMVd)	*Chrysanthemum* spp.	Conventional ^1^	SYBR Green I, gel electrophoresis, and real-time fluorescence	Lab protocol	Park et al. [117]
Chrysanthemum stem necrosis virus (CSNV)	*Dendranthema grandiflorum*; *Solanum lycopersicum*	RT-LAMP ^3^	Real-time fluorescence on a portable instrument	Lab protocol and crude method	Suzuki et al. [118]
Chrysanthemum stunt viroid (CSVd)	*Chrysanthemum morifolium*	Multiplex RT-LAMP ^3^	Gel electrophoresis coupled with EcoR I digestion; visual dyes	Lab kit	Liu et al. [119]
Chrysanthemum Virus B (CVB)	*Chrysanthemum morifolium*	Multiplex RT-LAMP ^3^	Gel electrophoresis coupled with EcoR I digestion; visual dyes	Lab kit	Liu et al. [119]
Cucumber green mottle mosaic virus (CGMMV)	*Cucurbitaceae*	RT-LAMP ^3^ and conventional ^1^	Gel electrophoresis and SYBR Green I	Lab protocol and kits	Bhat et al. [120]; Kwon et al. [121]
Cucurbit chlorotic yellows virus (CCYV)	*Bemisia tabaci*	RT-LAMP ^3^	Gel electrophoresis, SYBR Green I, and turbidity	Lab kit	Wang et al. [122]; Okuda et al. [123]
Grapevine leafroll-associated virus type 3 (GLRaV-3)	*Vitis* spp.	RT-LAMP ^3^	Gel electrophoresis, turbidity, and HNB	Lab protocol and crude extraction	Walsh et al. [124]
Leaf curl viral disease (CLCuD)	*Gossypium hirsutum*	Conventional ^1^ coupled with rolling circle amplification	Gel electrophoresis and visual dyes	Lab kit	Gawande et al. [125]
Little cherry virus 1 (LChV-1)	*Prunus* spp.	RT-LAMP ^3^	Real-time fluorescence on a portable instrument	Lab kit and crude method	Tahzima et al. [126]
Maize chlorotic mottle virus (MCMV)	*Zea mays*	RT-LAMP ^3^	Gel electrophoresis and visual dyes	Lab kit and crude method	Chen et al. [127]
Mesta yellow vein mosaic virus (MeYVMV)	*Hibiscus sabdariffa* and *H. cannabinus*	Conventional ^1^	Gel electrophoresis and HBN ^4^	Lab protocol	Meena et al. [128]
Onion yellow dwarf virus (OYDV)	*Allium cepa*	RT-LAMP ^3^	Real-time fluorescence, gel electrophoresis, and SYBR Green I	Lab kit	Tiberini et al. [129]
Papaya leaf distortion mosaic virus (PLDMV)	*Carica papaya*	RT-LAMP ^3^	Gel electrophoresis and SYBR Green I	Lab kit	Shen et al. [130]
Papaya ringspot virus (PRSV)	*Carica papaya*	RT-LAMP ^3^	Gel electrophoresis and SYBR Green I	Lab kit	Shen et al. [131]
Pea enation mosaic virus (PEMV)	*Pisum sativum*	Conventional ^1^	Gel electrophoresis and SYBR Green I	Lab kit	Kim et al. [132]
Pepper chat fruit viroid (PCFVd)	*Capsicum annuum*	RT-LAMP ^3^	Real-time fluorescence on a portable instrument and visual dyes	Lab kit and suitable field method	Tangkanchanapas et al. [133]
Piper yellow mottle virus (PYMoV)	*Piper nigrum*	Conventional ^1^ and RT-LAMP ^3^	Turbidity, calcein, and gel electrophoresis	Lab protocol	Bhat et al. [120]
Plum viroid I (PlVd-I)	*Harpephyllum caffrum*	RT-LAMP ^3^	Gel electrophoresis, visual dyes, and real-time fluorescence	CTAB ^6^-based and crude method	Bester and Maree, [134]
Potato Leafroll virus (PLRV)	*Solanum tuberosum*	Conventional ^1^	Gel electrophoresis and visual dyes	Lab protocol	Almasi et al. [135]
Potato virus a (PVA)	*Solanum tuberosum*	RT-LAMP ^3^	Gel electrophoresis and SYBR Green I	Lab protocol	Raigond et al. [136]
Prune dwarf virus (PDV)	*Prunus* spp.	RT-LAMP ^3^	Kit-based colorimetric visualization and gel electrophoresis	Lab kit	Çelik, [137]
Prunus necrotic ringspot virus (PNRSV)	*Prunus* spp.	RT-LAMP ^3^	Gel electrophoresis and SYBR Green I	Magnetic nanoparticle-based	Zong et al. [138]
Rice ragged stunt virus (RRSV)	*Oryza* spp.	RT-LAMP ^3^	Real-time fluorescence and gel electrophoresis	Lab kit	Lai et al. [139]
Sorghum mosaic virus (SrMV)	*Saccharum hybrids* spp.	RT-LAMP ^3^	Gel electrophoresis and SYBR Green I	CTAB ^6^-based	Keizerweerd et al. [140]
Southern tomato virus (STV)	*Solanum lycopersicum*	RT-LAMP ^3^	Gel electrophoresis and GelRed	Lab protocol	Elvira-González et al. [141]
Squash leaf curl virus (SLCV)	*Cucurbita pepo*; *Cucumis melo*	Conventional ^1^	Gel electrophoresis and SYBR Green I	Lab protocol	Kuan et al. [142]
Sugarcane streak mosaic virus (SCSMV)	*Saccharum hybrids* spp.	RT-LAMP ^3^	Gel electrophoresis and SYBR Green I	CTAB ^6^-based	Keizerweerd et al. [140]; Wang et al. [143]
Sweet potato feathery mottle virus (SPFMV), Sweet potato chlorotic stunt virus (SPCSV) and sweet potato leaf curl virus (SPLCV)	*Ipomoea batatas*	RT-LAMP ^3^	Kit-based colorimetric visualization and gel electrophoresis	CTAB ^6^-based	Wanjala et al. [144]
Telosma mosaic virus (TeMV)	*Passiflora edulis*	RT-LAMP ^3^	Gel electrophoresis and SYBR Green I	Lab protocol and kits	Fu et al. [145]
Tobacco streak virus (TSV)	*Gossypium hirsutum*	RT-LAMP ^3^	Gel electrophoresis, SYBR Green I, and HNB	Lab kit	Gawande et al. [146]
Tomato chlorosis virus (ToCV)	*Solanum lycopersicum*	RT-LAMP ^3^; conventional ^1^	Gel electrophoresis and SYBR Green I	Lab kit	Zhao et al. [147]; Kil et al. [148]
Tomato leaf curl Joydebpur virus (ToLCJoV)	*Capsicum anuum*	Conventional ^1^	Gel electrophoresis and HBN ^4^	Lab kit and crude method	Krishnan et al. [149]
Turnip yellows virus (TuYV)	*Brassica oleracea*; *B. napus*; *Cicer arietinum*; *Pisum sativum*; *Vicia faba*; *Lens culinaris*; *Lactuca sativa*; *Myzus persicae*	RT-LAMP ^3^	Real-time fluorescence on a portable instrument	Lab kit and crude method	Congdon et al. [150]
Ugandan cassava brown streak virus (UCBSV)	*Manihot esculenta*	RT-LAMP ^3^	Real-time fluorescence on a portable instrument and LFD ^5^	CTAB ^6^-based	Tomlinson et al. [112]
BACTERIA					
*Candidatus Liberibacter asiaticus* ^(IAPP)^	*Citrus* spp.	Conventional ^1^	Gel electrophoresis, SYBR Green I, real-time fluorescence, and LFD ^5^-combined	CTAB ^6^-based	Rigano et al. [151]; Wu et al. [152]
*Erwinia amylovora* ^(IAPP)^	More than 100 naturally infected samples from different hosts	Conventional ^1^	Real-time fluorescence on a portable instrument	Lab kit and boiling method	Bühlmann et al. [153]
*Pantoea ananatis*	*Oryza* spp.	Conventional ^1^	Kit-based colorimetric visualization	Lab kit	Kini et al. [154]
*Pectobacterium carotovorum* ^(IAPP)^	*Apium graveolens*	Conventional ^1^	Gel electrophoresis, calcein, and turbidity	Lab kit and crude method	Shi et al. [155]
*Xanthomonas euvesicatoria* ^(IAPP)^	*Solanum lycopersicum*	Based on assimilating probe	Real-time fluorescence and multiplex	Lab kit	Beran et al. [156]
*Xanthomonas fragariae* ^(IAPP)^	*Fragaria × ananassa*	Conventional ^1^	Real-time fluorescence on a portable instrument	Lab kit and crude method	Gétaz et al. [157]
*Xanthomonas oryzae pv. Oryzae* ^(IAPP)^	*Oryza* spp.	Conventional ^1^	Gel electrophoresis, GelRed, and real-time fluorescence on a portable instrument	Lab kit; CTAB ^6^-based	Buddhachat et al. [158]; Ejaz et al. [159]
*Xanthomonas phaseoli pv. Manihotis* ^(IAPP)^	*Manihot esculenta*	Conventional ^1^	Colorimetric dyes	Lab kit	Carvalho et al. [160]
*Xanthomonas vesicatoria* ^(IAPP)^	*Solanum lycopersicum*	Based on assimilating probe	Real-time fluorescence and multiplex	Lab kit	Beran et al. [156]
*Xylella fastidiosa* ^(IAPP)^	*Nerium oleander*; *Prunus avium*; *P. dulcis*; *Polygala myrtifolia*; *Acacia saligna*; *Olea europeae*; *Philaenus spumarius* and *Neophilaenus campestris*; *Vitis vinifera*; *Citrus sinensis*	Conventional ^1^; fluorescence of TaqMan Probe upon dequenching LAMP (FTP-LAMP); coupled with CRISPR-Cas12a	Gel electrophoresis, HBN ^4^, and real-time fluorescence on a portable instrument	Lab kit and suitable field methods; crude method	Aglietti et al. [1]; Harper et al. [161]; Yaseen et al. [162]; Elbeaino et al. [163]; Amoia et al. [164]; Farrall et al. [165]
PHYTOPLASMAS AND NEMATODES					
*Bogia Coconut Syndrome* *Phytoplasma* ^(IAPP)^	Insects	Conventional ^1^	Real-time fluorescence on a portable instrument	Lab kit	Lu et al. [166]
*Bursaphelenchus xylophilus* ^(IAPP)^	*Pinus* spp.	Conventional ^1^ and probe-based	Visual dyes and LFD ^5^	Lab kit	Kikuchi et al. [167]
*Flavescence dorée (FDp)* ^(IAPP)^	*Vitis* spp.	Conventional ^1^	Real-time fluorescence on a portable instrument	CTAB ^6^-based and on-site homogenization	Kogovšek et al. [168]
*Globodera pallida* ^(IAPP)^	Nematodes	Probe and microchip-based	Biochip platform measurement	Lab kit and Flinder Technology Associates (FTA) card-based	Camacho et al. [169]
*Meloidogyne hapla* ^(IAPP)^	Roots, adults, and eggs	Conventional ^1^	SYBR Green I dye, gel electrophoresis, and LFD ^5^	Combination with FTA technology	Peng et al. [170]
INSECTS					
*Agrilus planipennis* ^(IAPP)^	Adults, larvae, eggs, and *Fraxinus* spp. frass	Conventional ^1^	Real-time fluorescence on a portable instrument	Lab kit and crude method	Kyei-Poku et al. [171]
*Aromia bungii* ^(IAPP)^	Adults, larvae, and frass	Conventional ^1^	Real-time fluorescence, HBN ^4^, and gel electrophoresis	CTAB ^6^-based	Rizzo et al. [172]
*Bemisia tabaci* ^(IAPP)^	Adults	Conventional ^1^	SYBR Green I dye, gel electrophoresis, and real-time fluorescence on a portable instrument	Quick and on-site test	Dickey et al. [173]; Hsieh et al. [174]; Blaser et al. [175]
Fruit flies (Bactrocera and Zeugodacus genus)	Adults	Conventional ^1^	Real-time fluorescence on a portable instrument	On-site test	Blaser et al. [175]
*Lyctus brunneus*	Adults and wood frass	Conventional ^1^	Fluorescent dyes and gel electrophoresis	Lab kit	Ide et al. [176]
*Myzus persicae*	Individual aphids	Conventional ^1^	HBN ^4^ and gel electrophoresis	Crude method	Sial et al. [177]
*Pityophthorus juglandis* ^(IAPP)^	Walnut frass, adults, and larvae	Conventional ^1^	HBN ^4^, real-time fluorescence, and gel electrophoresis	Lab kit	Rizzo et al. [178]
*Spodoptera frugiperda* ^(IAPP)^	Larvae	Conventional ^1^	Real-time fluorescence on portable instrument	Lab kit and crude method	Congdon et al. [179]
*Thrips palmi* ^(IAPP)^	Adults and larvae	Conventional ^1^	Fluorescent dyes, gel electrophoresis, and real-time fluorescence on a portable instrument	Lab kit and on-site test	Przybylska et al. [180]; Blaser et al. [175]
*Trogoderma granarium* ^(IAPP)^	Adult and larvae	Conventional ^1^	Real-time fluorescence on a portable instrument	Lab kit and crude method	Rako et al. [181]

^1^ A conventional LAMP reaction as implemented by Notomi et al. [38] can contain Loop primers [182]. ^2^ RAPD markers = Random Amplification of Polymorphic DNA. ^3^ RT-LAMP: reverse transcript-LAMP used for RNA analysis. ^4^ HBN = hydroxy naphthol blue. ^5^ LFD = lateral-flow device test. ^6^ CTAB = Cetyltrimethylammonium Bromide. ^IAPP^ Invasive and/or regulated organism.

**Table 2:** Comparison of the effectiveness of PCR and LAMP for POC applications.

	Point-of-Care Features						
Technique	Approx. Time Needed	Cost per Analysis	Thermal Cycle Required	Portable Instruments Availability	Work with Unprocessed Samples	Simplified DNA/RNA Extraction	Advanced Lab Skills Required
PCR	>1 h	USD 12 (end-point PCR)	Yes	Yes but expensive	No	No	Yes
LAMP	From 30 min to 1 h	USD 3	No, isothermal reaction	Yes, including low-cost solutions	Yes	Yes	No

## Data Availability

No new data were created or analyzed in this study.
